# P-691. Characteristics Associated with the Presence of One or More Risk Factors for Severe Respiratory Syncytial Virus Disease among Adults in the United States

**DOI:** 10.1093/ofid/ofae631.887

**Published:** 2025-01-29

**Authors:** Emily K Horn, David Singer, Alison Booth, Cynthia Saiontz-Martinez, Ariel Berger

**Affiliations:** GSK, Philadelphia, Pennsylvania; GSK, Philadelphia, Pennsylvania; Evidera PPD, Basel, Basel-Stadt, Switzerland; Evidera PPD, Basel, Basel-Stadt, Switzerland; Evidera PPD, Basel, Basel-Stadt, Switzerland

## Abstract

**Background:**

Respiratory syncytial virus (RSV) can cause severe disease in older adults and adults with certain medical conditions. In June 2023, RSV vaccination was recommended for United States (US) adults ≥60 years of age (YoA) using shared clinical decision-making. This study explored factors associated with having diagnosed and undiagnosed risk factors (RFs) for severe RSV disease among US adults.

**Figure:**
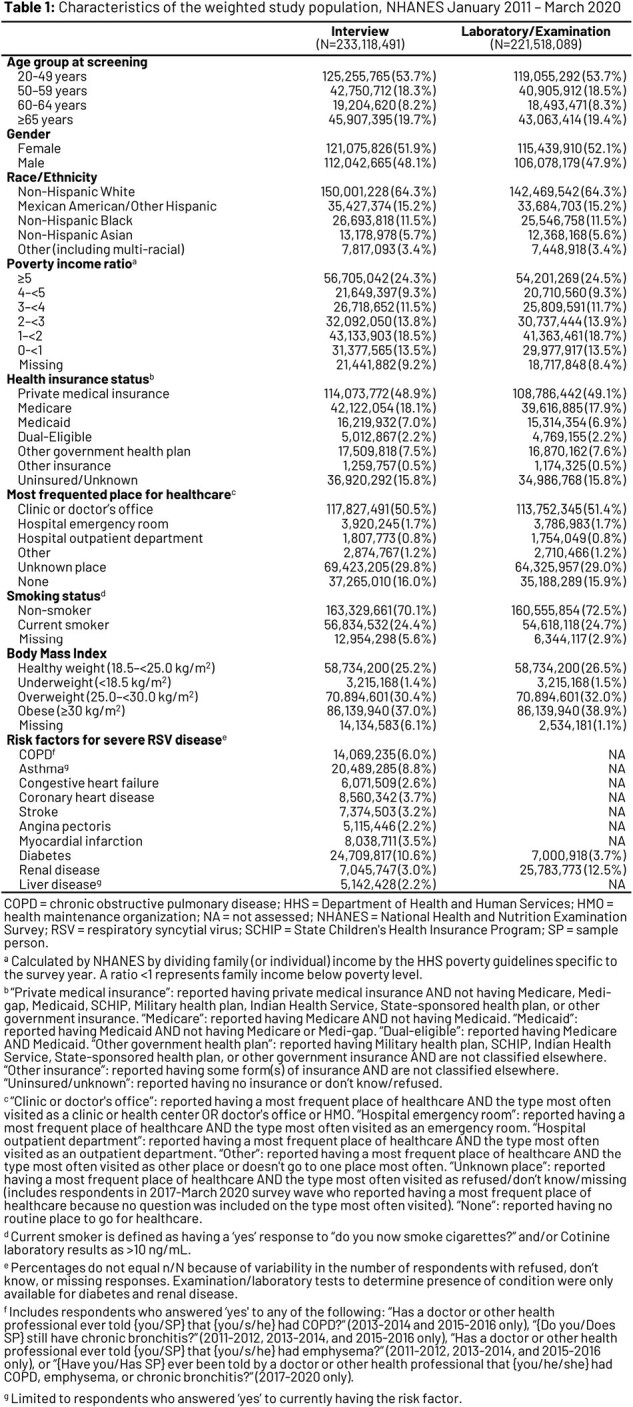

**Methods:**

A retrospective analysis of pooled National Health and Nutrition Examination Survey data from 4 survey waves (January 2011 - March 2020) was conducted, comprising adult respondents (≥20 YoA), and weighted to allow extrapolation to all non-institutionalized US adults (**Table 1**). Diagnosed RFs were identified via self-report; undiagnosed RFs were assessed via lab test/exam results (limited to diabetes and renal disease). Multivariable logistic regression models were developed to assess associations between respondents’ sociodemographic characteristics and presence of ≥1 RF for severe RSV disease (cardiopulmonary, endocrine, and metabolic conditions).

**Figure:**
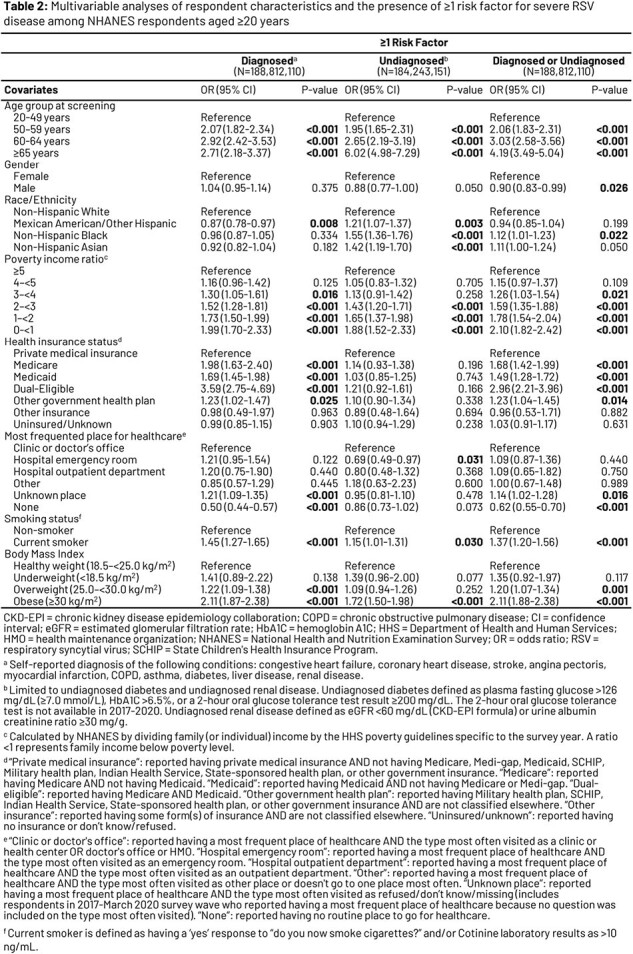

**Results:**

After extrapolation to the US population, 35.5% of adults ≥20 YoA (n=82,715,916/233,102,301) had ≥1 RF (diagnosed or undiagnosed) for severe RSV disease. For both diagnosed and undiagnosed RFs, older age, lower income, being a smoker, and obesity were associated with significantly higher odds of having ≥1 RF (**Table 2**). Adults with government insurance had a higher likelihood of having ≥1 diagnosed RF compared with those with private insurance (p < 0.05). Adults from racial and ethnic minority groups were more likely than non-Hispanic White adults to have ≥1 undiagnosed RF (p < 0.01). Compared to adults whose routine place for healthcare was doctors’ offices, adults with no routine place were less likely to have ≥1 diagnosed RF (p < 0.001), while those whose routine place was the emergency room were less likely to have ≥1 undiagnosed RF (p < 0.05).

**Conclusion:**

Among US adults, several social determinants of health are associated with the presence of ≥1 diagnosed and undiagnosed RF for severe RSV disease. Understanding characteristics associated with RFs for severe RSV disease can help to ensure RSV vaccination among these higher risk groups.

**Funding:** GSK (GSK study identifier: VEO-000686)

**Disclosures:**

**Emily K. Horn, MSc**, GSK: employee|GSK: Stocks/Bonds (Private Company) **David Singer, PharmD, MS**, GSK: employee|GSK: Stocks/Bonds (Public Company) **Alison Booth, MSc**, GSK: Grant/Research Support|Other pharmaceutical, biotech, and medical device companies: Grant/Research Support **Cynthia Saiontz-Martinez, MSc**, GSK: Grant/Research Support|Other pharmaceutical, biotech, and medical device companies: Grant/Research Support **Ariel Berger, MPH**, GSK: Grant/Research Support|Other pharmaceutical, biotech, and medical device companies: Grant/Research Support

